# Overexpression of IL‐10 in Adipose Mesenchymal Stem Cells Promotes Wound Healing in Diabetic Mice

**DOI:** 10.1155/sci/8861898

**Published:** 2026-01-28

**Authors:** Hui Zhao, Feng Song, Long Ouyang, Xiao Shi, Shuai Shang

**Affiliations:** ^1^ Plastic Surgery Hospital, Chinese Academy of Medical Sciences and Peking Union Medical College, Beijing, 100144, China, cacms.ac.cn; ^2^ Department of Clinical Laboratory, The Affiliated Taian City Central Hospital of Qingdao University, Tai’an, 271600, China, qdu.edu.cn; ^3^ Department of Critical Care Medicine, Shandong Provincial Hospital Affiliated to Shandong First Medical University, Jinan, China, sph.com.cn; ^4^ Department of Equipment Section, Taian 88 Hospital, Tai’an, 271600, China

**Keywords:** ADSC, diabetic ulcer, IL-10, overexpression, wound healing

## Abstract

**Objective:**

Diabetic ulcers are serious chronic wounds that are challenging to heal and can lead to amputation or even death. This study aims to utilize interleukin‐10 (IL‐10) overexpressing adipose mesenchymal stem cells to investigate their potential in promoting the healing of diabetic ulcers and to explore their mechanism of action.

**Methods:**

The analysis of stem cell characteristics of ADSC‐IL10 was performed through flow cytometry, cell scratch assay, MTT assay, and adipogenic and osteogenic differentiation assays. The detection of the M1 and M2 phenotypes of mouse peritoneal macrophages (RAW 264.7) under conditioned medium stimulation was carried out using qPCR technology. The assessment of the effects of conditioned media from ADSCs overexpressing IL‐10 (ADSC‐IL10 CM) and conditioned media from adipose‐derived stem cells (ADSC CM) on the migration of normal skin fibroblasts and human immortalized epidermal cells was done using Transwell and cell scratch methods. A diabetic mouse model was induced using a high‐fat/high‐sugar diet plus streptozotocin (STZ) to detect the number of M2 macrophages and the expression levels of inflammatory factors (IL‐1*β*, IL‐6, IL‐10, and MCP‐1) and growth factors (EGF, VEGF, and TGF*β*‐1) in mouse skin tissue.

**Results:**

The overexpression of IL‐10 did not change the biological properties of ADSCs. In diabetic mice, the transplantation of IL‐10 overexpressing ADSCs for wound healing was more effective than the transplantation of ADSCs alone. ADSCs overexpressing IL‐10 promoted the expression of M2 macrophages marker; inhibited the secretion of proinflammatory factors such as IL‐1*β*, IL‐6, and MCP‐1; and enhanced the production of growth factors including EGF, TGF*β*‐1, and VEGF. Furthermore, it facilitated the migration of skin fibroblasts and epidermal cells from diabetic mice to the wound site.

**Conclusion:**

ADSCs that overexpress IL‐10 promote wound healing in diabetic mice by reducing inflammatory responses, enhancing growth factor secretion, and increasing the migration of fibroblasts and epidermal cells.

## 1. Introduction

Diabetic foot ulcers are a significant complication of diabetes, frequently resulting in amputation or even mortality [1–3]. As the global incidence of diabetes continues to escalate, the life expectancy of those with diabetes is also increasing, which in turn leads to a rising incidence and mortality rate of diabetic foot ulcers. This trend imposes an increasingly substantial economic burden on society and healthcare systems [4, 5]. The mechanism underlying chronic wounds is intricate and remains incompletely understood. Inflammation plays a crucial role in the healing process, with the orderly migration of neutrophils and macrophages being essential for effective wound repair. Within the wound environment, these cells secrete a variety of inflammatory mediators that orchestrate the healing cascade [6]. Consequently, for the successful completion of wound repair and tissue regeneration, the body’s inflammatory response must be meticulously regulated. Interleukin‐10 (IL‐10), a key regulatory factor in suppressing inflammatory responses, has demonstrated significant effects. Studies indicate that IL‐10 inhibits the activation of proinflammatory cytokines in macrophages, suppresses various chemokines, and reduces leukocyte migration to inflamed sites [7–12]. It also regulates the JAK‐STAT signaling pathway to alleviate fibrosis [13–18]. Furthermore, IL‐10 serves as a crucial regulatory factor in tissue repair, playing a pivotal role in limiting and terminating inflammatory responses during this process [11, 19].

Stem cells have been extensively researched for their potential to accelerate wound healing. Patients suffering from advanced conditions, such as diabetic ulcers or deep chronic wounds, who have no alternative but amputation, may benefit from stem cell therapy [13, 20–25]. Studies have demonstrated that BM‐MSCs, MSCs, and their extracellular matrix (ECM) can serve as novel therapeutic agents with antiapoptotic, anti‐inflammatory, and antifibrotic effects [26–28]. Additionally, MSCs promote cell proliferation and migration, thereby facilitating bone regeneration [29, 30]. However, BMSCs are derived from bone marrow, and their availability is limited. Derived from adipose tissue, adipose‐derived stem cells (ADSCs) have been proven to provide a basis for regeneration after tissue injury by regulating immune response and to promote wound healing through paracrine action [31–36]. ADSCs, due to their ease of procurement compared to BMSCs and their abundant supply, have increasingly become the cell type of choice for wound repair and regeneration, garnering significant interest from medical professionals and researchers alike. In this study, we aimed to investigate whether ADSCs expressing IL‐10 through overexpression could accelerate diabetic wound healing by reducing inflammatory responses and promoting cell proliferation. Our findings demonstrate that ADSC‐IL10 accelerates diabetic wound healing by suppressing inflammatory responses (through promoting macrophage M2 polarization) and enhancing the expression of growth factors such as EGF, TGF*β*‐1, and VEGF.

## 2. Materials and Methods

### 2.1. Experimental Animals and Experimental Cells

The ADSCs utilized in the experiments were exclusively sourced from liposuction patients at the Plastic Surgery Hospital of the Chinese Academy of Medical Sciences, aged between 20 and 35 years. The motile cells, mouse peritoneal macrophages (RAW 264.7 cells), were obtained from the Basic Medical Cell Center of the Institute of Basic Medical Sciences at the Chinese Academy of Medical Sciences. The fibroblasts originated from surgical patients at the Plastic Surgery Hospital of the Chinese Academy of Medical Sciences. The HEK 293T cells were sourced from the research center of the Plastic Surgery Hospital of the Chinese Academy of Medical Sciences. The experimental animals included SPF grade 8‐week‐old Balb/c mice, with weights ranging from 24 g to 28 g, all procured from Beijing Vital River Laboratory Animal Technology Co., Ltd. The experimental animals were housed in the standardized animal room of the Plastic Surgery Hospital of the Chinese Academy of Medical Sciences. All experimental procedures were approved by the Ethics Committee of Plastic Surgery Hospital, Chinese Academy of Medical Sciences.

### 2.2. Real‐Time PCR Primer Sequences

The RUNX family transcription factor 2 (RUNX2) was utilized as a marker for osteogenic differentiation, whereas peroxisome proliferator‐activated receptor *γ* (PPAR*γ*) served as a marker for adipogenic differentiation. CD68 was employed to detect the mRNA expression of M1 macrophages in RAW 264.7 cells and tissues on day 7 posttrauma in mice. Similarly, arginase‐1 (Arg‐1) and CD206 were used to assess the mRNA expression of M1 macrophages in RAW 264.7 cells and tissues on day 7 posttrauma in mice. The inflammatory levels of RAW 264.7 cells and mouse wound cells were gauged using IL‐1*β*, IL‐6, IL‐10, and MCP‐1 as indicators, while the expression levels of growth factors in mouse wounds were measured using EGF, TGF*β*‐1, and VEGF as markers. The genes, primer sequences, sequence numbers, and coding DNA lengths used in the experiments are detailed in Table 1.

**Table 1:** Sequence, serial number, and DNA length coding of genes and primers used in the experiment.

Gene	Serial number	Primer sequence	Length (bp)
RUNX2	NM_001024630.3	F: ACTGGCGCTGCAACAAGAC R: CCCGCCATGACAGTAACCA	91
PPAR*γ*	NM_138711.3	F: TGGAATTAGATGACAGCGACTTGG R: CTGGAGCAGCTTGGCAAACA	182
Arg‐1	NM_007482.3	F: AATGAAGAGCTGGCTGGTGT	153
		R: AGTGTGAGCATCCACCCAAA	
CD206	NM_008625.2	F: CACGGAGATCCACGAGCAAA	131
		R: GATACCGGAATGGGCTTCCT	
CD86	NM_019388.3	F: GCAGGACCAGCAAAAGTTGG	151
		R: CGAGCCCATGTCCTTGATCT	
IL‐10	NM_010548.2	F: CAGAGAAGCATGGCCCAGAA	129
		R: GCTCCACTGCCTTGCTCTTA	
IL‐1*β*	NM_008361.4	F: ATGCCACCTTTTGACAGTGATG	136
		R: TGTGCTGCTGCGAGATTTGA	
IL‐6	NM_001314054.1 NM_031168.2	F: TCCGGAGAGGAGACTTCACA	167
		R: TTCTGCAAGTGCATCATCGT	
MCP‐1	NM_011333.3	F: ACCTGCTGCTACTCATTCACC	148
		R: ATTCCTTCTTGGGGTCAGCA	
VEGF	NM_001110268.1 NM_001110267.1	F: CTTCGAGGAGCACTTTGGGT	141
		R: CCCTAATCTTCCGGGCTTGG	
EGF	NM_001329594.1 NM_010113.4	F: CATGGAGACAGAAGCCCCAC	126
		R: CCACAGGTCTGTAGGAGGGT	
TGF*β*‐1	NM_011577.2	F: CGTCAGACATTCGGGAAGCA	140
		R: ACCAAGGTAACGCCAGGAAT	
GAPDH	NM_002046.7	F: GCACCGTCAAGGCTGAGAAC R: TGGTGAAGACGCCAGTGGA	138

### 2.3. Isolation and Cultivation of ADSC and Fibroblasts

ADSC: The adipose tissue suspension obtained from liposuction was first washed twice with sterile PBS. Transfer 20 mL of tissue suspension into a 50 mL centrifuge tube and then add an equal volume of 0.2% type I collagenase. Digest for 50 min using a 37 °C incubator shaker. After digestion, centrifuge the adipose tissue first at 1200 rpm for 5 min, followed by centrifugation with an equal volume of mesenchymal stem cell medium at 1200 rpm for 5 min. Add 10 mL of mesenchymal stem cell medium and mix thoroughly. Filter through a 100‐mesh cell sieve and then transfer the filtrate to a cell culture incubator for cultivation.

Fibroblasts: Surgical skin tissue should be immersed in 75% alcohol for 2 min. Following this, wash the tissue with PBS containing penicillin‐streptomycin (P/S) for a minimum of three cycles. Remove adipose and connective tissues using ophthalmic scissors. Cut the skin samples into 0.5 × 0.5 cm segments, place them in dishes containing a 0.1% dispase solution, seal with cellophane film, and incubate overnight in a 4 °C refrigerator. The next day, dissect the epidermis from the dermis using forceps. Mince the debris and transfer it to centrifuge tubes. Digestion should proceed in a 37 °C water bath with 0.1% type I collagen, 0.1% type II collagen, and 0.1% type IV collagen for 2 h. Filter the mixture through a 100 μm mesh and centrifuge 300 g aliquots for 5 min. Resuspend the precipitates in human dermal fibroblast complete medium, plate them, and culture in a 37 °C incubator.

### 2.4. Conditioned Media From ADSCs (ADSC CM) and ADSC CM Overexpressing IL‐10 (ADSC‐IL10 CM)

When the ADSCs/ADSC‐IL10 cells reach 80% confluence, remove the initial medium and replace it with 10 mL of serum‐free, antibiotic‐free DMEM low‐sugar medium. Incubate the culture. After a 24‐h cultivation period, collect the ADSCs/ADSC‐IL10 conditioned culture medium (ADSC CM and ADSC‐IL10 CM). Transfer 50 mL of the conditioned medium into centrifuge tubes, filter it through a 0.22 μm filter, and store the filtered medium at −20 °C for subsequent use.

### 2.5. Flow Cytometry

The cell density of the ADSCs was adjusted to 5 × 10^6^ cells/mL. Samples were incubated with CD 44, CD 73, CD 90, and CD 105 antibodies (Human Mesenchymal Stem Cell Analysis Kit, BD Biosciences, Table 2) for 30 min. One milliliter of PBS was added to each tube to remove excess antibodies, followed by centrifugation for 5 min. Cell surface markers were detected using a FACS Aria II flow cytometer (BD Biosciences). The results were analyzed using FlowJo 10 software (Ashland, OR, USA).

**Table 2:** Proportion of stem cell markers identified in ADSCs by flow cytometry.

EP tube number	Antibody name	Adding volume (μL)
1	FITC Mouse Antihuman CD90	5
2	PE Mouse Antihuman CD44	5
3	PerCP‐Cy5.5 Mouse Antihuman CD105	5
4	APC Mouse Antihuman CD73	5
5	Nothing	—
6	hMSC Positive Isotype Control Cocktail PE hMSC Negative Isotype Control Cocktail	20 20
7	hMSC Positive Cocktail PE hMSC Negative Cocktail	20 20
8	hMSC Positive Isotype Control Cocktail Drop in isotype control (i.e., PE Mouse IgG2b, *κ*)	20 5
9	hMSC Positive Cocktail PE Drop in (i.e., PE Mouse Antihuman CD44)	20 5

### 2.6. Fibroblast and Epidermal Cell Migration Experiment

Fibroblast migration: Add 120 μL of the fibroblast suspension to the 8 μm Transwell chamber (upper chamber) and culture in a 5% CO2 incubator for 18 h. In a new 24‐well plate, add 4% tissue cell fixation solution, place the upper chamber in the fixative, and fix at room temperature for 30 min. Stain with crystal violet, rinse, and observe the cells under a microscope for photography. Transfer the chamber membrane to a 96‐well plate containing 100 μL of 30% acetic acid, incubate for 10 min, and then place the acetic acid in a spectrophotometer to detect the absorbance at a wavelength of 570 nm for analysis.

Epidermal cell migration: Uniformly draw vertical lines at the bottom of a 6‐well plate; then inoculate HaCat cells and draw three horizontal lines uniformly on the cells at the bottom of the 6‐well plate. The ADSC CM group is added with 2 mL of ADSC CM, and the control group is added with 2 mL of DMEM medium containing 2% serum. Photographs are taken at fixed positions on the scratch areas at 0, 6, and 12 h, respectively.

### 2.7. Proliferation Experiment of ADSC Cells

Prepare the cell suspension and inoculate the appropriate number of cells into a 96‐well plate for plating. Incubate in a 5% CO2 incubator for 4 h. Add 10 µL of CCK8 to each well and incubate for an additional 2 h in a 5% CO2 incubator. Measure the absorbance at 450 nm using a microplate reader. Use GraphPad Prism 7 to plot the proliferation curve of ADSC‐IL10 cells based on the absorbance values.

### 2.8. Induce ADSCs to Differentiate Into Adipose/Bone In Vitro

The recovered ADSCs were inoculated into 6‐well plates, with 2 mL of complete ADSC medium added to each well and cultured in a 37 °C incubator. Upon achieving complete cell confluence, the medium was discarded and replaced with 2 mL of osteoinductive mesenchymal stem cell (OMSC) adipogenesis medium. After 7 days of adipogenesis induction, the medium was removed, the plate was washed twice with PBS, and the triol reagent was added to extract total RNA for subsequent detection of adipogenesis marker genes. Oil red O staining was performed 7 days after adipogenesis induction to evaluate the in vitro adipogenic efficacy of ADSC‐IL10.

The ADSC‐IL10 cells were seeded into 6‐well plates precoated with 0.1% gelatin, with 2 mL of complete ADSC medium added to each well. The cells were cultured in a 37 °C incubator, with the medium changed every 2 days. For adult adipose‐derived mesenchymal stem cells, 2 mL of complete osteogenic differentiation medium was added. After 14 days of osteogenic induction, the differentiation medium was discarded, and the cells were washed twice with PBS before trichloroacetic acid was used to extract total RNA for subsequent detection of osteogenic marker genes. Following 21 days of osteogenic differentiation induction using complete osteogenic differentiation medium, the ADSC‐IL10 cells were stained with carmine red to evaluate the effectiveness of osteogenic induction.

### 2.9. Diabetes Mouse Model

Male SPF BALB/c mice, aged 8 weeks, were housed in an animal facility and fed a high‐fat, high‐sugar diet for five consecutive days. After completing this feeding regimen, the mice underwent a 12‐h fasting period with water deprivation, followed by an intraperitoneal injection of streptozotocin (STZ) at a dose of 150 mg/kg. The control group received an equivalent dose of sodium citrate buffer solution (0.1 mol/L and pH 4.5) instead of STZ. Both groups resumed their respective diets postinjection and maintained them until the end of the study. Following three consecutive days of STZ administration, random blood glucose levels were monitored for 14 days. Starting from the fourth day of injections, three consecutive random blood glucose tests exceeding 14.1 mmol/L and fasting blood glucose levels above 11.1 mmol/L were required to confirm successful diabetes modeling. Mice with blood glucose levels exceeding 16.7 mmol/L were selected for formal experimental participation.

### 2.10. Construction of a Diabetic Mouse Trauma Model

Forty‐five hyperglycemic mice, with blood glucose levels exceeding 16.7 mmol/L, were selected for the formal experiment. They were then randomized into three groups: the control group, the ADSC‐PCDH group, and the ADSC‐IL10 group, with 15 mice in each. In the ADSC‐PCDH and ADSC‐IL10 groups, subcutaneous transplantation of ADSC‐PCDH cells and ADSC‐IL10 cells, respectively, was performed on the back wounds of the mice, with ~1 × 10^6^ cells transplanted into each mouse. The control group received an injection of 0.9% normal saline. The transplanted mice were housed in individual ventilated cages (IVCs) for further care and were monitored on days 1, 3, 5, 7, and so forth, during which time photographs were taken of the back wounds of the mice.

### 2.11. Statistical Analysis

All measurement data were expressed as mean ± standard deviation (SD), and the *t*‐test was used to analyze whether there were statistical differences between groups. A *p*‐value of less than 0.05 indicated that the differences between groups were statistically significant. The results were calculated using GraphPad Prism 7, and a histogram was drawn. The wound area of mice was calculated using ImageJ. Photoshop CS6 was utilized to merge immunofluorescence images. The results of qPCR and flow cytometry were analyzed using FlowJo.

## 3. Results

### 3.1. The Overexpression of IL‐10 Did Not Affect the Proliferation, Migration, or Differentiation of ADSCs

The culture media for ADSC‐IL10, ADSC‐PCDH, and ADSC cells were prepared, and the secretion of IL‐10 in the supernatant was measured using ELISA. No significant variation in IL‐10 expression was observed between the ADSC and ADSC‐PCDH groups (*n* = 3, *p* = 0.6591), In contrast, a significant difference was noted between the ADSC‐IL10 group and the ADSC‐PCDH group (*n* = 3, *p*  < 0.0001; Figure 1A). Cell surface markers, including CD44, CD73, CD90, and CD105, were analyzed on ADSC, ADSC‐PCDH, and ADSC‐IL10 cells via flow cytometry. The findings indicated that CD44, CD73, CD90, and CD105 were all positive in the ADSC, ADSC‐PCDH, and ADSC‐IL10 groups (Figure 1B).

Figure 1Characteristics of IL‐10 overexpression in ADSC. (A) Expression of IL‐10 in cell supernatant. (B) Flow cytometry demonstrated that most ADSCs expressed CD44/CD73/CD90 and CD105. (C) Overexpression of IL‐10 had no effect on ADSC proliferation. (D) Overexpression of IL‐10 had an effect on ADSC migration. (E) Overexpression of IL‐10 had no effect on adipogenic differentiation ADSC. (F) Overexpression of IL‐10 had no effect on osteogenic differentiation of ADSC. ns indicates significant difference;  ^∗^^∗^^∗^^∗^ indicates that the difference is extremely significant (*p*  < 0.0001).

**Figure:**
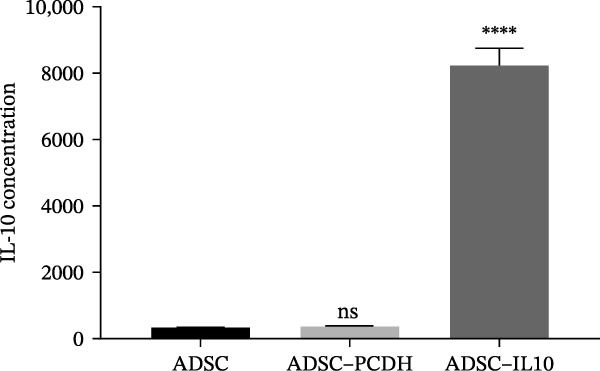
(A)

**Figure:**
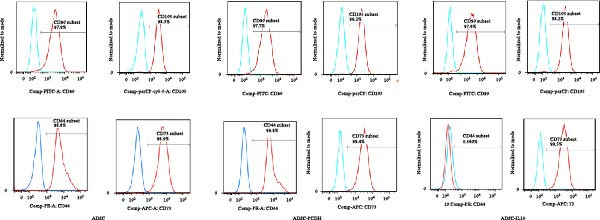
(B)

**Figure:**
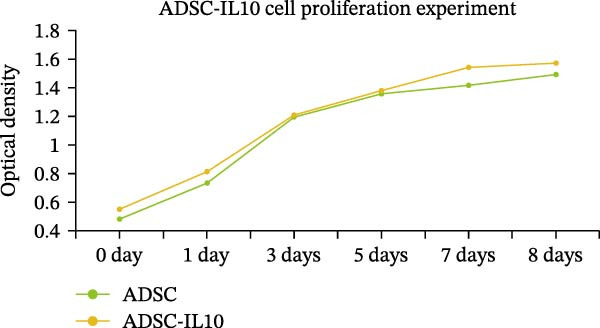
(C)

**Figure:**
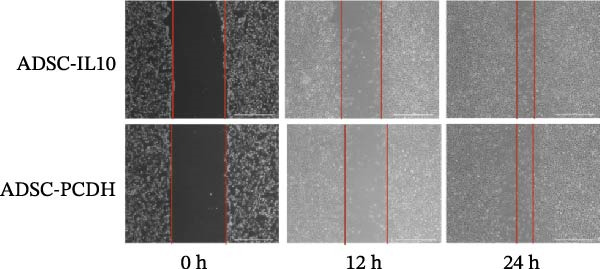
(D)

**Figure:**
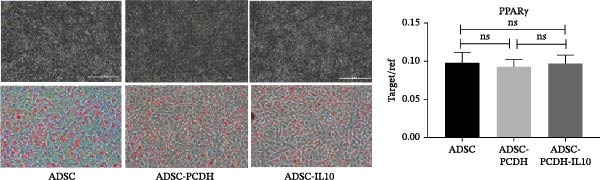
(E)

**Figure:**
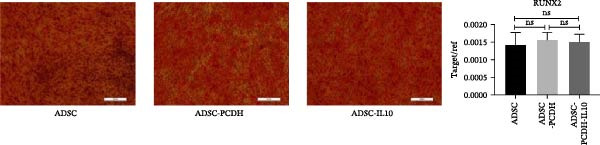
(F)

The MTT assay was utilized to assess the cellular proliferation of both the ADSC‐PCDH and ADSC‐IL10 groups. The outcomes indicated no significant disparities in cell proliferation between the two groups, as their proliferation curves exhibited a relatively uniform pattern (Figure 1C). The examination of cell migration within the ADSC‐PCDH group and the ADSC‐IL10 group disclosed no significant difference in migratory activity between the two cohorts. Both groups demonstrated complete cell migration by the 24‐h mark, with no statistically significant variation in the number of migrated cells (Figure 1D).

Adipogenic differentiation was induced in P5 ADSCs, ADSC‐PCDH cells, and ADSC‐IL10 cells. A significant accumulation of lipid droplets was observed under the microscope on the fifth day of lipid induction. Oil red O staining indicated that ADSC, ADSC‐PCDH, and ADSC‐IL10 cells all had equivalent adipogenic differentiation potential. Total RNA was isolated from ADSC, ADSC‐PCDH, and ADSC‐IL10 cells on the fifth day, and the expression of the lipid‐producing marker gene PPAR*γ* was assessed using quantitative polymerase chain reaction (qPCR). The results showed that there was no statistically significant difference in the expression of the PPAR*γ* gene among the three groups (*n* = 3; Figure 1E).

Osteogenic differentiation was induced in P5 ADSC, ADSC‐PCDH, and ADSC‐IL10 cells. On the 21st day of osteogenic induction, these cells were stained with alizarin red. Microscopic examination revealed that ADSC, ADSC‐PCDH, and ADSC‐IL10 cells exhibited equivalent staining capabilities with alizarin red. Total RNA was extracted from ADSC, ADSC‐PCDH, and ADSC‐IL10 cells on day 14, and the expression of the osteogenic marker RUNX2 was quantified using qPCR. The outcomes indicated that there was no significant difference in RUNX2 expression between ADSC‐IL10 and ADSC‐PCDH cells (*p* = 0.7638), nor between ADSC‐PCDH cells and the control group (*n* = 3, *p* = 0.6072), or between ADSC‐IL10 cells and the control group (*n* = 3, *p* = 0.7744; Figure 1F).

### 3.2. The Overexpression of IL‐10 in ADSCs Enhances the Acceleration of Chronic Wound Healing in Diabetic Mice

The diabetic mice were randomly assigned to one of three groups: control, ADSC‐PCDH, or ADSC‐IL10. A wound measuring 1.5 cm by 1.5 cm was excised from the dorsum, and PBS, 1 × 10^6^ ADSC‐PCDH cells, and 1 × 10^6^ ADSC‐IL10 cells were subcutaneously transplanted into each group, respectively. It was observed that the healing rate of diabetic mice in the ADSC‐IL10 group (with wounds healed by day 19) was significantly higher than that in the ADSC‐PCDH group (healed by day 21) and the control group (healed by day 25). Mice in the control group exhibited nonhealing wounds (Figure 2A).

Figure 2Characteristics of wound healing in diabetic mice. (A) Characteristics of wound surface in diabetic mice at days 0, 3, 7, 10, 14, and 19. (B) Comparison of healing area between control/ADSC‐PCDH/ADSC‐IL10 group, *n* = 3.  ^∗^ indicates *p*  < 0.05;  ^∗∗^ indicates *p*  < 0.01;  ^∗∗∗^ indicates *p*  < 0.001;  ^∗^^∗^^∗^^∗^indicates *p*  < 0.0001.

**Figure:**
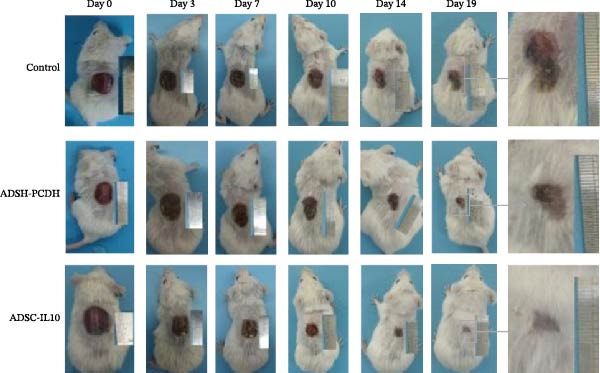
(A)

**Figure:**
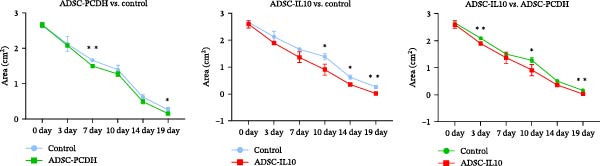
(B)

ImageJ was utilized to evaluate the wound area in mice, and the results revealed a significant difference in wound size between the ADSC‐IL10 group and the ADSC‐PCDH group on the third day of healing (*p* = 0.0026). By the seventh day of healing, a notable difference in wound size became apparent between the ADSC‐PCDH group and the control group (*p* = 0.0097). Upon reaching the tenth day of healing, both the ADSC‐IL10 group and the ADSC‐PCDH group displayed significant differences in wound size compared to each other (*p* = 0.0457) and to the control group (*p* = 0.0240). By the fourteenth day of healing, the ADSC‐IL10 group exhibited a significant difference in wound size compared to the control group (*p* = 0.0156). Finally, on the nineteenth day of healing, significant differences in wound size were observed between the ADSC‐IL10 group and the ADSC‐PCDH group (*p* = 0.0013), the ADSC‐IL10 group and the control group (*p* = 0.0027), as well as between the ADSC‐PCDH group and the control group (*p* = 0.0364; Table 3; Figure 2B).

**Table 3:** Statistics of wound area in diabetic mice (*n* = 3).

Group	0 day (cm^2^)	3 day (cm^2^)	7 day (cm^2^)	10 day (cm^2^)	14 day (cm^2^)	19 day (cm^2^)
Control	2.65 ± 0.046	2.12 ± 0.121	1.66 ± 0.029	1.39 ± 0.069	0.61 ± 0.045	0.26 ± 0.034
ADSC‐PCDH	2.65 ± 0.036	2.08 ± 0.027	1.49 ± 0.021	1.27 ± 0.050	0.48 ± 0.042	0.15 ± 0.009
ADSC‐IL10	2.59 ± 0.080	1.89 ± 0.006	1.36 ± 0.115	0.90 ± 0.119	0.35 ± 0.048	0.02 ± 0.013

*Note:* The data in the table are mean (x) ± standard deviation (SD).

### 3.3. The Transplantation of ADSCs Overexpressing IL‐10 Facilitated the Polarization of Macrophages Towards the M2 Phenotype

On the seventh day postwounding, granulation tissues from diabetic mice were subjected to CD68 and Arg‐1 dual fluorescence staining. CD68 served as a marker for all macrophages (both M1 and M2 types), while Arg‐1 represented a secretory protein specific to M2 macrophages. The findings indicated that the expression of Arg‐1 in the skin of diabetic mouse wound grafts treated with ADSC‐IL10 was elevated compared to those treated with ADSC‐PCDH and the control group, exhibiting a statistically significant difference. Although there was an increase in Arg‐1 expression in the ADSC‐PCDH transplanted group relative to the control group, this difference was not statistically significant. These results suggest that ADSC‐IL10 can enhance the population of M2–type macrophages within the wounds of diabetic mice (Figure 3A).

Figure 3At 7 days, M2 macrophages in the wound tissue. (A) Arg‐1 in wound tissue image. (B) The expression of Arg‐1 and CD206 gene in wound tissue, *n* = 3.  ^∗^ indicates *p*  < 0.05;  ^∗∗^ indicates *p*  < 0.01;  ^∗∗∗^ indicates *p*  < 0.001;  ^∗^^∗^^∗^^∗^ indicates *p*  < 0.0001.

**Figure:**
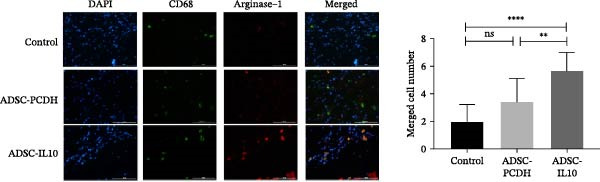
(A)

**Figure:**
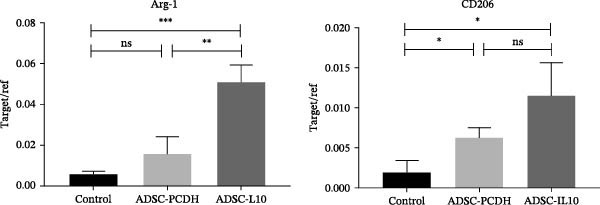
(B)

On the seventh day postwounding in diabetic mice, the RNA expression levels of two M2 macrophage markers, namely, Arg‐1 and CD206, were assessed using qPCR in the newly formed granulation tissue. The investigation revealed that the RNA expression levels of both Arg‐1 and CD206 were significantly elevated in the wounds of diabetic mice that received ADSC‐IL10 transplants compared to those that received ADSC‐PCDH transplants and the control group. Statistically significant differences were observed. Additionally, the expression of CD206 RNA in the wounds of diabetic mice from the ADSC‐PCDH group was also found to be higher than that in the control group, indicating a statistical difference. These findings suggest that ADSC‐IL10 transplantation can enhance the expression of M2–type macrophages at the RNA level within the wounds of diabetic mice (Table 4; Figure 3B).

**Table 4:** Arg‐1/CD206 RNA qPCR target/reference values in wound tissues of diabetic mice on day 7 (*n* = 3).

Group	Arg‐1	CD206
Control	0.00598 ± 0.000813	0.001975 ± 0.0008421
ADSC‐PCDH	0.01573 ± 0.004788	0.006327 ± 0.0006684
ADSC‐IL10	0.0512 ± 0.004693	0.01155 ± 0.002329

*Note:* CD206 and Arg‐1 are M2 macrophage markers; the data in this table are mean (x) ± standard deviation (SD).

### 3.4. The Transplantation of ADSCs That Overexpress IL‐10 Enhanced the Expression of Anti‐Inflammatory and Growth Factors at the Wound Site

Granulation tissues from diabetic mice were collected on the seventh day postwounding, and total RNA was subsequently extracted. The expression levels of inflammatory cytokines IL‐1*β*, IL‐4r, and IL‐10 were assessed using reverse transcription and fluorescence‐based real‐time quantitative PCR (*n* = 3). The findings indicated that in the wounds of diabetic mice that received ADSC‐IL10 transplants, there was a reduction in the expression of the proinflammatory cytokine IL‐1*β* RNA compared to the control group. Conversely, the expression of the anti‐inflammatory cytokines IL‐4r and IL‐10 RNA was elevated, with statistically significant differences observed. In contrast, no marked variation in the expression of IL‐1*β* and IL‐4r was noted between the ADSC‐PCDH transplanted group and the control group. The data presented in Figure 4A suggest that ADSC‐IL10 transplantation may attenuate the inflammatory response on the wound surface more effectively than ADSC‐PCDH transplantation.

Figure 4Expression levels of inflammatory cytokines and growth factors in wound tissue. (A) Expression of IL‐1*β*, IL‐4R, and IL‐10 in wound tissue, *n* = 3. (B) Expression of EGF, TGF*β*‐1, and VEGF in wound tissue, *n* = 3.  ^∗^ indicates *p*  < 0.05;  ^∗∗^ indicates *p*  < 0.01;  ^∗∗∗^ indicates *p*  < 0.001;  ^∗^^∗^^∗^^∗^indicates *p*  < 0.0001.

**Figure:**
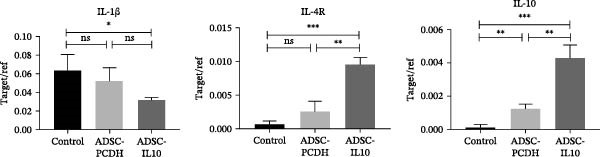
(A)

**Figure:**
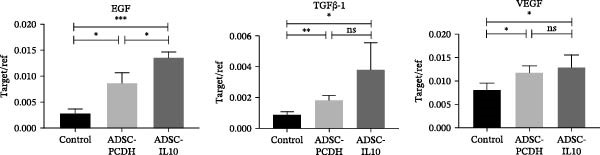
(B)

Granulation tissues from diabetic mice were collected on the seventh day postwound, and total RNA was subsequently extracted. The expression levels of EGF, TGF*β*‐1, and VEGF RNA were assessed using reverse transcription and fluorescence‐based real‐time quantitative PCR. The findings indicated that the RNA expression levels of EGF, TGF*β*‐1, and VEGF in the wounds of diabetic mice within the ADSC‐IL10 and ADSC‐PCDH groups were comparable to those observed in the control group. No statistically significant differences were noted in the expression levels of TGF*β*‐1 and VEGF RNA between the ADSC‐IL10 and ADSC‐PCDH groups, suggesting a pivotal role for ADSC cells (Figure 4B).

### 3.5. ADSC‐IL10 CM Induces the Differentiation of RAW 264.7 Cells Into M2–Type Macrophages

ADSC‐IL10 CM and ADSC CM were collected to stimulate the peritoneal macrophages of mice, and the RNA of RAW 264.7 cells was extracted to detect the expression levels of CD86, CD206, and Arg‐1 mRNA (*n* = 3). The results indicated that both ADSC‐IL10 CM and ADSC CM induced a significant increase in the expression of M2–type macrophage markers CD206 and Arg‐1 in RAW 264.7, while ADSC‐IL10 CM also suppressed the expression of the M1–type macrophage marker CD86 in RAW 264.7. These findings suggest that ADSC‐IL10 CM facilitates the differentiation of RAW 264.7 cells towards an M2 phenotype (Figure 5A).

Figure 5Conditioned medium stimulated the RAW 264.7 cell. (A) Expression of CD86, CD206, and Arg‐1 in RAW 264.7 cell, *n* = 3. (B) Under the stimulus of LPS + IFN*γ*, the expression of IL‐1*β*, IL‐6, and MCP‐1 in RAW 264.7 cell, *n* = 3. (C) Expression of EGF, TGF*β*‐1, and VEGF in RAW 264.7 cell, *n* = 3.  ^∗^ indicates *p*  < 0.05;  ^∗∗^ indicates *p*  < 0.01;  ^∗∗∗^ indicates *p*  < 0.001;  ^∗^^∗^^∗^^∗^ indicates *p*  < 0.0001.

**Figure:**
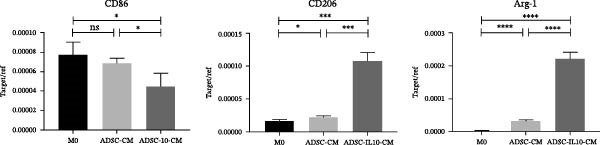
(A)

**Figure:**
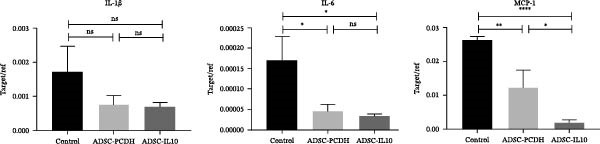
(B)

**Figure:**
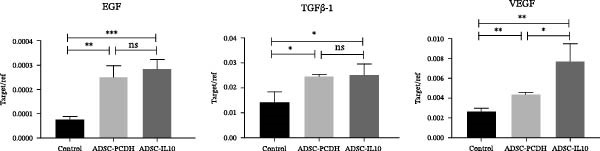
(C)

RAW 264.7 cells were induced into an inflammatory state by stimulation with lipopolysaccharide (LPS) and interferon gamma (IFN*γ*). Subsequently, conditioned media from ADSC CM and ADSC‐IL10 CM were introduced. After 12 h of stimulation, RNA was extracted from the RAW 264.7 cells to assess the expression levels of proinflammatory cytokines, including interleukin‐1 beta, interleukin‐6, and monocyte chemoattractant protein‐1 mRNA (*n* = 3). The findings indicated that the addition of ADSC‐IL10 CM led to a decrease in the expression of these proinflammatory cytokines. This implies that ADSC‐IL10 CM has the potential to suppress the inflammatory response in RAW 264.7 cells (Figure 5B).

ADSC‐IL10 CM and ADSC CM were collected to stimulate RAW 264.7 cells, respectively. Subsequently, RNA was extracted from the RAW 264.7 cells to assess the expression levels of EGF, TGF*β*‐1, and VEGF mRNA (*n* = 3). The findings indicated that following the culture of RAW 264.7 cells with both ADSC‐IL10 CM and ADSC CM, there was an upregulation in the expression of EGF, TGF*β*‐1, and VEGF mRNA. Notably, the upregulation was more pronounced with ADSC‐IL10 CM (Figure 5C).

### 3.6. ADSC CM Facilitates the Migration of Fibroblasts and Epidermal Cells

The migration of normal skin fibroblasts was investigated using conditioned media from ADSCs, with and without IL‐10 supplementation (*n* = 3). The findings indicated that, in contrast to DMEM, both ADSC CM and ADSC‐IL10 conditioned media facilitated the migration of fibroblasts. The *p*‐values for these effects were 0.0078 and 0.0314, respectively. Furthermore, the ADSC‐IL10 conditioned media group exhibited a greater number of migrated cells than the ADSC–conditioned media group; however, this difference was not statistically significant (Figure 6A). Furthermore, HaCat cells were cultured in the presence of ADSC CM and DMEM medium supplemented with 2% serum, and the migration of HaCat cells was assessed. The findings indicated that the migration of HaCat cells cultured with ADSC CM was essentially complete by 12 h, whereas the migration rate of those cultured in DMEM medium with 2% serum was notably slower, exhibiting a significant difference compared to the ADSC CM group (Figure 6B).

Figure 6Effects of conditioned medium on fibroblast and epidermal cell migration. (A) Effects of ADSC CM and ADSC‐IL10 CM on fibroblast migration. (B) Effect of ADSC CM on HaCat cell migration.  ^∗^ indicates *p*  < 0.05;  ^∗∗^ indicates *p*  < 0.01;  ^∗∗∗^ indicates *p*  < 0.001;  ^∗^^∗^^∗^^∗^ indicates *p*  < 0.0001.

**Figure:**
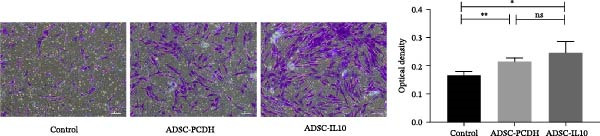
(A)

**Figure:**
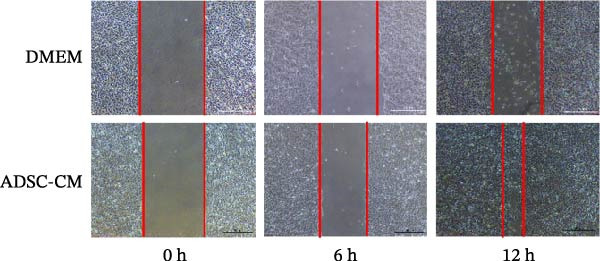
(B)

## 4. Discussion

The impaired healing of diabetic ulcers is characterized by a persistent inflammatory state that disrupts the normal tissue regeneration process, largely due to aberrant cytokine production [37]. In this study, we pioneered a combinatorial therapeutic strategy by engineering adipose‐derived mesenchymal stem cells (ADSCs) to overexpress the potent anti‐inflammatory cytokine IL‐10. Our findings demonstrate that the transplantation of ADSC‐IL10 into the wounds of diabetic mice significantly accelerated wound healing compared to both unmodified ADSC‐PCDH and control treatments. Specifically, wounds in the ADSC‐IL10 group achieved complete closure within 19 days, a notable improvement over the ADSC‐PCDH group (21 days) and the control group (25 days). This enhanced efficacy underscores the synergistic potential of combining stem cell therapy with targeted immunomodulation.

A critical factor in the pathogenesis of chronic wounds is the dysregulation of macrophage polarization, where a predominance of proinflammatory M1 macrophages over reparative M2 macrophages hinders healing. IL‐10, a key cytokine produced by Th2 cells and regulatory T cells, is known for its ability to suppress inflammatory responses and promote M2 polarization, thereby facilitating tissue repair and ECM remodeling [38]. However, the clinical application of IL‐10 is impeded by its short half‐life in wound environments [39]. Our approach, which involves using ADSCs as sustained delivery vehicles for IL‐10, effectively circumvents this limitation. We observed a significant increase in M2 macrophages at the wound site on day 7 posttransplantation in the ADSC‐IL10 group, accompanied by a favorable cytokine profile characterized by the downregulation of proinflammatory genes (IL‐1*β* and IL‐6) and the upregulation of anti‐inflammatory IL‐10. This confirms that ADSC‐IL10 alleviates the inflammatory blockade in diabetic wounds by driving macrophage polarization towards the beneficial M2 phenotype.

Beyond the contribution of IL‐10, ADSCs themselves played a vital role in promoting repair through their well‐documented paracrine functions, including immunomodulation and the secretion of growth factors. The accelerated healing observed in the ADSC‐PCDH group compared to the control validates the therapeutic capacity of ADSCs alone. However, the superior performance of the ADSC‐IL10 group highlights a critical enhancement. The mechanistic distinction likely lies in the more robust induction of M2 macrophages and a stronger anti‐inflammatory milieu (characterized by elevated IL‐4R and IL‐10) in the ADSC‐IL10 group, which creates a more conducive environment for subsequent reparative phases.

The transition from inflammation to proliferation is crucial for wound closure. Our results indicated a significant upregulation of key growth factors—EGF, TGF*β*‐1, and VEGF—in wounds treated with ADSC‐IL10. These factors play a crucial role in facilitating the migration and proliferation of keratinocytes and fibroblasts and also in stimulating angiogenesis [40]. The inadequate blood supply typical of diabetic wounds poses a major obstacle to healing. Thus, the enhanced expression of VEGF, in particular, likely improved neovascularization and nutrient delivery to the wound site. The synergistic action of these growth factors, facilitated by ADSC‐IL10 treatment, would significantly accelerate re‐epithelialization and tissue remodeling.

In conclusion, our study demonstrates that the overexpression of IL‐10 in ADSCs does not compromise their inherent biological properties; instead, it enhances their therapeutic potential for diabetic wound healing. The mechanism involves a multifaceted approach: suppressing detrimental inflammation through M2 macrophage polarization, increasing the expression of pivotal growth factors, and facilitating fibroblast migration. While this study provides compelling evidence for the efficacy of ADSC‐IL10, limitations persist. The precise mechanisms underlying ADSC–alone–mediated healing and the potential for ADSC differentiation into adipose tissue in vivo require further investigation. First, the long‐term safety of ADSC‐IL10 transplantation has not been evaluated, which is a core issue that must be addressed before clinical application. Second, there are differences between mouse and human diseases, so the results should be cautiously extrapolated to clinical practice. Future research will also focus on elucidating the specific pathways by which ADSC‐IL10 promotes macrophage M2 polarization. Nonetheless, this combinatorial strategy presents a promising and novel avenue for the treatment of recalcitrant diabetic ulcers.

## Author Contributions

Hui Zhao and Feng Song designed and directed the experiments. Long Ouyang, Xiao Shi, and Shuai Shang performed the experiments. Hui Zhao and Feng Song analyzed the data and wrote the manuscript.

## Funding

This work was supported by Tai’an Science and Technology Development Innovation Project (Grant 2021NS392).

## Disclosure

All authors read and approved the final manuscript.

## Ethics Statement

The authors have nothing to report.

## Consent

All content of this study has been approved and standardized by the Medical Ethics Committee of the Plastic Surgery Hospital (Number 202066), Chinese Academy of Medical Sciences.

## Conflicts of Interest

The authors declare no conflicts of interest.

## Data Availability

The data generated or analyzed during the study are included in this article, and datasets are available from the corresponding author upon reasonable request.
